# Adolescents’ experiences of the information they received about the coronavirus (Covid-19) in Norway: a cross-sectional study

**DOI:** 10.1186/s13034-021-00384-4

**Published:** 2021-06-16

**Authors:** Sabine Kaiser, Henriette Kyrrestad, Monica Martinussen

**Affiliations:** Regional Center for Child and Youth Mental Health-North (RKBU-North), Faculty of Health Sciences, UiT The Arctic University of Norway, 9037 Tromsø, Norway

**Keywords:** Adolescents, Information, Coronavirus, Covid-19, Pandemic

## Abstract

**Background:**

In the first months of the coronavirus (Covid-19) pandemic, many countries took radical prevention measures. Authorities had to communicate with the public regularly to explain and ensure compliance with these measures and promote safety. The information given by authorities was mainly developed for adults, but children and adolescents may have different needs when it comes to information. This study examined how adolescents perceived information about Covid-19 provided by the media and other sources, and about what topics adolescents reported they lacked information during the first months of the Covid-19 pandemic.

**Methods:**

Three hundred seventy-seven adolescents in 11th, 12th, and 13th grade in Norwegian upper secondary schools (67% girls) completed an online questionnaire. Analyses included descriptive statistics, in addition to Generalized Linear Mixed Models analyses to examine gender differences in adolescents’ satisfaction with the information provided about Covid-19, to what extent the pandemic affected their everyday life, and to what extent they were concerned about becoming infected with Covid-19.

**Results:**

The results showed that the majority of adolescents used the internet as the main source of information about Covid-19, followed by (online) newspapers. About half (49%) reported that they were satisfied with the information available, while 39% were neither satisfied nor dissatisfied, and 12% were dissatisfied. Adolescents wanted more information about the school situation, as well as virus- and future-related topics. A total of 21% reported that they were concerned about becoming infected with Covid-19. There was no significant gender difference in overall satisfaction with the information provided about Covid-19. Girls reported being significantly more affected by the pandemic than boys, and that they were significantly more concerned than boys about becoming infected with Covid-19.

**Conclusions:**

This survey provides important knowledge to professionals working with adolescents, as well as to authorities, about what information channels can be used to reach adolescents, and what information they lacked about the Covid-19 pandemic.

## Background

Efficient dissemination of information is an important part of public health work, and it is especially important in exceptional situations, like the one that arose during the coronavirus (Covid-19) pandemic. A report from the Norwegian Institute of Public Health found that communicating information about Covid-19 plays a huge role in reducing infections, and places a low to no burden on children and adolescents [1]. Nevertheless, a survey of the adult population in Norway showed that “a significant proportion of the population faces a variety of challenges in dealing with health information” (p. 12) [2]. If this is true for adults, how do adolescents perceive the information they receive about Covid-19?

Covid-19 is a flu-like respiratory infection that, in January of 2020, started to spread from China to many countries around the world. Covid-19 is transmitted through droplets and close contact, and appears to have a higher mortality rate than the flu, making it a priority to reduce its spread among the population [3]. The World Health Organization declared Covid-19 a “serious incident of significance to international public health” in January 2020, and in March 2020 it was declared a pandemic. Many European countries, including Norway, implemented radical measures to reduce the spread of Covid-19 in the population. These measures were similar to those taken in 107 other countries, and included closing kindergartens and schools [4]. People who had traveled abroad or between southern and northern Norway, and those with flu-like symptoms were obligated to quarantine at home for 2 weeks. Anyone who was not considered an essential worker (i.e., who performed tasks critical to society) was encouraged to work from home, and students across Norway received online teaching only.

The Norwegian health authorities provided official recommendations and information to the public several times per day through the media, through the Norwegian Institute of Public Health’s websites, and through the governmental website Helsenorge.no. These recommendations were comprehensive and targeted various groups, such as at-risk groups, pregnant women and children, persons in quarantine, persons with symptoms, health care personnel, as well as other occupational groups. The recommendations changed over time as the situation developed, and many experts commented on the situation in the media, sometimes with viewpoints that contradicted the official recommendations of health authorities.

Given the importance of the successful dissemination of information in public health, it is useful to look at the characteristics that contribute to this success. Some have argued that, for its dissemination to be successful, information has to be applicable, diverse, reliable, and comprehensible [5]. Among other things, applicable information refers to information that is relevant for tackling a health issue and appropriate to local practice. Diverse information targets a variety of people who might have different needs (such as children, adolescents, adults, pregnant women, and older people). Reliable information is based on evidence [5] and enables the individual to better protect him/herself and thus experience a sense of mastery of the situation. A Chinese study concluded that “erroneous knowledge could lead to ineffective preventive measures by the population and increase the risk of infection” (p. 302) [6]. Finally, to be comprehensible, information must be easily understood by its recipients [5].

Most information provided during the pandemic targeted adults [7] and was probably challenging for children and adolescents to navigate [8]. Indeed, key messages need to be adapted in an age-appropriate way. Not only might children and adolescents be interested in different topics than adults, but research suggests that adolescents process information differently than adults, as their brains are still developing [9–11]. Furthermore, girls and boys in adolescence may understand and interpret information differently. Girls generally have better reading skills than boys [12], which may impact both the speed at which they read and their comprehension of written information about the virus. One study found small gender differences in mental health literacy among adolescents [13]. Females were able to recognize and label mental health problems more correctly than males, and the prevalence of a disorder within a gender was related to the recognition of that disorder by the gender [13]. Bjørnsen et al. [14] also found small gender differences in mental health literacy, but they concluded that overall differences might be smaller than once assumed.

Information provided to children and adolescents must be applicable, reliable, and comprehensible, but it must also be tailored to their age group. The Norwegian authorities were aware of this, and held a special press conference on March 16th 2020, during which the Prime Minister, Erna Solberg, the Minister of Children and Families, and the Minister of Education and Integration answered questions that children had submitted. No similar press conference was held for adolescents, whose daily lives have been affected by the pandemic in unprecedented ways. Indeed, being confined to their homes with limited social contact may have a bigger impact on adolescents than on younger children and adults [15, 16]. Adolescence is a period in which many explore different options related to vocation, relationships, and other areas before making commitments [17], and this was not possible in traditional ways during the pandemic. Moreover, adolescence is a developmental period, when information might be obtained, understood, and handled differently than in other age groups [18]. Most adolescents do not develop severe symptoms when infected with Covid-19 [19], which might make then less inclined to seek out information due to a lower perceived risk [20]. One study found that higher perceived risk among adolescents was associated with the presence of risk factors for and knowledge of Covid-19, and that knowledge was related to better compliance with preventive measures [21], suggesting that improving knowledge about Covid-19 could be a good strategy to increase protective behavior in this age group [21]. Abbott et al. [20] criticized messaging efforts aimed at adolescents in the USA, which stressed that it is unlikely the virus would make them seriously ill. They argued that adolescents may have been more motivated to engage in protective behaviors if they were reminded about the possible consequences of infection for people in high-risk groups, such as older people [20]. Yang et al. [21] came to the same conclusion, and found that protecting family and friends at risk was a motivating factor for adolescents to adhere to preventive measures.

Adolescents may also be less likely to follow guidelines because they seek greater autonomy; they may prefer to listen to friends for guidance and information, instead of listening to more reliable information from adults or official institutions [22]. The potential consequences of adolescents not seeking information or following guidelines may be a public health risk, as they could spread the virus to more vulnerable populations. Disseminating effective information tailored to adolescents could help promote safe behaviors and prevent the spread of Covid-19 [23]. It is therefore important to examine how adolescents perceived the information provided on Covid-19 through the media and other sources.

Results from an international study showed that 61% of Norwegian 9th graders used traditional media channels, such as TV and newspapers, to stay informed about Covid-19 [24]. The Norwegian Media Authority’s annual report (2020) showed that many children and adolescents read news on social media, and that proportion increased with increasing age [25]. The same report also showed that more girls than boys read, watch, and listen to news in general. A total of 52% of 9–10-year-olds and 96% of 15–18-year-olds get their daily news from social media [25]. YouTube, Snapchat, TikTok, and Instagram were the most popular social media platforms among those aged 9–18 years [25]. Whether these same platforms are preferred in a time of crisis is unknown. A survey based on approximately 1300 individuals, aged 16 years and above, showed that confidence in the Norwegian media is generally high [26]. However, it is not known whether adolescents have high confidence in the media when it comes to information about the Covid-19 pandemic. In general, trust can be divided into trust in other people and trust in institutions. The latter is important, as it also includes trust in the government, the municipality, the judiciary, the police, and the media [24]. In a previous survey, Norwegian 9th graders (*N* = 6271) had overall high confidence in Norwegian authorities [24]. A Swiss study found that trust in authorities was associated with compliance with Covid-19-related public health measures [27].

### Study aim

This study examined how adolescents perceived information about Covid-19 provided by the media and other sources, and about what topics adolescents reported they lacked information during the first months of the Covid-19 pandemic. Another aim of the study was to identify which social media platforms and media adolescents used to gather information, and how they evaluated the reliability and utility of these sources. In addition, we examined how adolescents perceived the Covid-19 pandemic, and how they were affected by the measures taken. Other aims were to examine gender differences in the overall satisfaction with information provided about Covid-19, in the extent to which the pandemic affected everyday life, and in concerns about becoming infected with Covid-19.

## Methods

### Participants and procedure

An email with information on the background and purpose of the survey, and a study link, was sent to the principals of all upper secondary schools in the two northernmost counties of Norway (Troms and Finnmark, number of schools = 22 and Nordland, number of schools = 16) on Monday March 16th 2020. Compared to other Norwegian counties, the two northernmost counties are characterized by a large geographical area and fewer inhabitants compared to the southernmost counties of Norway. The principals were asked to forward the link to the students at their school, and a reminder was sent to principals after 1 week. The head of school affairs in Nordland County declined to participate in any kind of surveys during this period. Of the 22 principals from Troms and Finnmark County, five shared the link with their students. These five schools varied in size (from 180 to 500 students) and in the educational curriculums offered (e.g., health and youth development, information technology and media production, sales, service and tourism, technology and industry subjects, crafts, design and product development, and building and construction).

The link was also published in a news article on the websites of the Regional Centre for Child and Youth Mental Health and Child Welfare and of UiT The Arctic University of Norway, as well as on the Faculty of Health Sciences at UiT The Arctic University of Norway Facebook page. The link took participants to a website, which presented information about the survey, and informed them that participation was voluntary and anonymous. Adolescents were not offered any type of incentive to complete the survey, which was administered using Nettskjema, a secure tool for online data collection. Since the survey was conducted anonymously and without obtaining detailed background information, it was not necessary to report the project to the NSD—Norwegian Centre for Research Data.

A total of 407 adolescents completed the online survey. Findings from a smaller sample of 354 adolescents from 8 to 13th grade, in addition to apprentices, who completed the survey between March 16th and March 26th 2020 were presented in a preliminary report in Norwegian [28]. The current article presents findings for 377 adolescents in 11th, 12th, and 13th grade, in addition to apprentices, who completed the survey between March 16th and May 5th 2020 (full study period). Adolescents in 8th, 9th, and 10th grade were excluded from the present analysis (*n* = 30) because they were not the target group of our study.

### Survey

The survey consisted of 15 questions. The first three collected information on demographic variables (county of residence, gender, and grade). There were two questions on sources of information. One asked adolescents what sources they used to find information about Covid-19, and which sources they believed had the best information. There were 15 response options (e.g., internet, school, radio, Table 1), and more than one option could be chosen.

**Table 1:** Sources adolescents used to get information about the coronavirus pandemic, and which sources they believed had the best information (multiple answers were possible; *N* = 377)

Response categories	Main sources of information		Best information	
	*n*	%	*n*	%
Internet	292	78	133	35
(Online) newspaper	243	65	156	41
TV	226	60	122	32
Family	210	56	59	16
Facebook	162	43	24	6
Friends	141	37	15	4
School	126	33	42	11
Homepage from the Public Health Institute	106	28	115	31
Snapchat	88	23	21	6
Homepage from the Directorate of Health	85	23	90	24
Helsenorge.no	79	21	68	18
Radio	72	19	26	7
TikTok	48	13	9	2
Other	19	5	14	4
Brochures, booklets, other material	8	2	5	1

Seven questions evaluated adolescents’ perception of the information provided about Covid-19 (e.g., Do you find the information provided easy to understand?, Table 2). Responses were given on a five-point scale (1 = not at all; 5 = to a very great extent). One question was used to evaluate adolescents’ satisfaction with the information provided about Covid-19 (Table 3), and responses were once again given on a five-point scale (1 = very dissatisfied; 5 = very satisfied).

**Table 2:** Adolescents’ perception of the information provided about the coronavirus pandemic (*N* = 370–375)

Variable	*M* (*SD*)	Not at all/to a small extent		To some extent		To a great extent/to a very great extent	
		*n*	%	*n*	%	*n*	%
Do you feel that the provided information is sufficient?	3.52 (0.93)	37	10	143	39	190	51
Do you find the provided information easy to understand?	3.70 (0.93)	35	9	109	29	231	62
Do you find the information you need about the situation?	3.64 (0.95)	40	11	123	33	209	56
All in all, I get the information I need	3.77 (0.99)	40	11	96	26	237	64
To what extent do you trust the provided information?	3.63 (0.86)	31	8	130	35	214	57
The provided information makes me feel safer	3.14 (1.07)	87	23	158	42	130	35
The provided information calms me down	2.86 (1.06)	127	34	157	42	89	24

The response categories “not at all” (1) and “to a small extent” (2) in addition to “to a large extent” (4) and “to a very large extent” (5) were combined

**Table 3:** Satisfaction with the information provided about the coronavirus pandemic, impact on everyday life, and concerns about infection (*N* = 374)

Variable	*M* (*SD*)	Dissatisfied/very dissatisfied		Neither		Satisfied/very satisfied	
		*n*	%	*n*	%	*n*	%
Overall, how satisfied or dissatisfied are you with the provided information about the coronavirus?	3.43 (0.87)	45	12	147	39	182	49

	*M* (*SD*)	Not at all/to a small extent		To some extent		To a great extent/to a very great extent	
		*n*	%	*n*	%	*n*	%
To what extent does this situation affect your everyday life?	3.96 (1.02)	35	9	69	18	271	72
To what extent are you concerned about getting infected with the coronavirus?	2.57 (1.16)	194	52	102	27	78	21

The response categories “very dissatisfied” (1) and “dissatisfied” (2) in addition to “satisfied” (4) and “very satisfied” (5) were combined. Also, the response categories “not at all” (1) and “to a small extent” (2) in addition to “to a large extent” (4) and “to a very large extent” (5) were combined

There were two questions regarding the impact the Covid-19 pandemic had on adolescents’ lives. One asked to what extent the pandemic affected their everyday life, and the other to what extent they were concerned about becoming infected with Covid-19 (Table 3). Response options were given a five-point scale (1 = not at all; 5 = to a very great extent). At the end of the survey, adolescents could list topics on which they wanted more information, using an open answer field.

### Statistical analyses

SPSS 26 was used to perform the analyses. In order to simplify the presentation of frequency distributions, response categories 1 and 2 (e.g., not at all and to a small extent) and 4 and 5 (e.g., to a great extent and to a very great extent) on the five-point scales were merged. Generalized Linear Mixed Models analyses were conducted without random effects, to examine gender (girl and boy) differences, controlling for grade [11th grade, 12th grade, 13th grade, and other (e.g., apprentice)], in the three dependent variables: overall satisfaction, effect on everyday life, and concern about becoming infected with Covid-19.

## Results

Of the 377 adolescents included in the analysis, 33% were boys and 67% were girls; 126 were in grade 11 (33%), 129 were in grade 12 (34%), 100 were in grade 13 (27%), and 22 were classified as other (e.g., apprentice 6%). Most participants were from the county Troms and Finnmark (*n* = 238; 63%), followed by Nordland (*n* = 53; 14%), Innlandet (*n* = 25; 7%), Vestland (*n* = 24; 6%), Agder and Viken (*n* = 5; 1%, respectively), Trøndelag (*n* = 4; 1%), Vestfold og Telemark (*n* = 3; 1%), and Rogaland (*n* = 2; 1%).

### Sources of information

The main sources of information about Covid-19 used by adolescents were the internet (78%) followed by (online) newspapers (65%), TV (60%), and their family (56%). Ratings showed that adolescents believed the best information was provided by (online) newspapers (41%), the internet (35%), and TV (32%), but the homepages of the Public Health Institute and the Directorate of Health also received high rankings (31% and 24%, respectively).

### Perception of and satisfaction with information, impact on everyday life, and concerns about infection

Most adolescents perceived the information provided about Covid-19 to be sufficient and understandable and stated that they trusted this information and felt that they got the information they needed. However, few adolescents reported that the provided information made them feel safer or that it calmed them down (Table 2).

Adolescents’ overall satisfaction with the information provided about Covid-19 showed that almost half (49%) were satisfied or very satisfied with it, while 12% were dissatisfied or very dissatisfied, and 39% stated that they were neither satisfied nor dissatisfied. A total of 72% found that the pandemic affected their everyday life to a great extent or to a very great extent, while the majority of adolescents (52%) was not concerned about becoming infected with Covid-19 (Table 3).

### Gender differences

Table 4 presents the results of the analyses used to examine gender differences after controlling for grade. Grade was not significant in any of the three models. There were no significant differences in overall satisfaction with the information provided about Covid-19 between boys and girls (*F*(1, 24) = 1.27, *p* = 0.27). Girls were significantly more affected by the pandemic than boys (*F*(1, 24) = 10.26, *p* < 0.01), and they were significantly more concerned about becoming infected with Covid-19 than boys (*F*(1, 24) = 9.12, *p* < 0.01).

**Table 4:** Generalized Linear Mixed Models Analyses to examine gender differences (*N* = 372)

Variable	Satisfaction with information		Affected by situation		Concerns about getting infected	
	B (SE)	Odds ratio (95% CI)	B (SE)	Odds ratio (95% CI)	B (SE)	Odds ratio (95% CI)
Grade						
Other^a^	− 0.24 (0.49)	0.79 (0.29; 2.17)	0.55 (0.47)	1.73 (0.66; 4.54)	0.29 (0.49)	1.34 (0.49; 3.69)
13th^a^	0.32 (0.30)	1.38 (0.75; 2.55)	0.20 (0.27)	1.22 (0.70; 2.11)	− 0.05 (0.27)	0.95 (0.54; 1.67)
12th^a^	0.08 (0.27)	1.08 (0.61; 1.90)	0.22 (0.25)	1.24 (0.74; 2.09)	0.22 (0.26)	1.25 (0.74; 2.12)
Gender						
Girl^b^	− 0.28 (0.25)	0.76 (0.46; 1.26)	0.71 (0.22)**	2.04 (1.29; 3.24)	0.70 (0.23)**	2.02 (1.25; 3.26)

**p < 0.01

^a^11th grade is the reference group

^b^Boys are the reference group

### Lack of information

A total of 60 of the 377 adolescents wrote in specific questions or statements on topics about which they wanted more information. These were entered into an Excel file and sorted according to thematic content by the third author. This resulted in three, approximately equal, categories, which were reviewed by the other authors. Disagreements regarding the categorization were resolved by discussion between the authors and only applied to a few items. The first category comprised questions related to school and exams; the second dealt with questions about Covid-19, infection, and disease; and the last category was related to opinions on information and questions about the future. Within the school category, many had questions related to exams and how the situation would affect the next school year. There were also apprentices who had lost weeks of school and had questions related to this. Some also mentioned that school had an important social function in their lives, and that this was now gone.

In the category of questions about Covid-19, infection, and disease, many had questions about the quarantine, and about who comprised the high-risk groups mentioned in the media. The latter was often justified by a concern for other family members. Some also wondered how far the science had come regarding finding a cure or vaccine for Covid-19.

The last category dealt with information and questions about the future. Some adolescents thought there was almost too much information in the media, and that this might frighten people more, while others did not think that the information was aimed at adolescents and their situation. Other questions in this category were about how long the pandemic would last, and what significance it would have for individuals, but also for Norway, the economy, and job opportunities.

## Discussion

### Sources of information

The results of this study showed that adolescents received information from many different sources during the first months of the Covid-19 pandemic in Norway. Internet, newspapers/online newspapers and TV, but also the family, were rated as the main sources of information about Covid-19. The traditional media channels such as TV and newspapers/online newspapers were received to the same extent as reported in a previous Norwegian study [24]. Adolescents considered that the best information was provided in newspapers/online newspapers and on the internet. Sources such as TV and the websites of the Norwegian Institute of Public Health and the Directorate of Health were preferred by many. Social media platforms, such as Facebook, were also used by many, but they were not considered a good source of information. This seems contrary to common beliefs that young people prefer social media, and to earlier findings from Norway indicating that almost all adolescents aged 15–18 years get their news from social media [25]. It seems that, when it comes to important information, traditional channels like newspapers (paper and online) are preferred. As mentioned before, health information provided to the public should be applicable, diverse, reliable, and comprehensible for a wider audience, including adolescents [5]. That means that information providers from more traditional channels should be aware that they also attract younger age groups in some situations, and the information they present should be adapted accordingly.

### Perception of and satisfaction with the information

Over half of the adolescents perceived the information provided about Covid-19 to be sufficient, understandable, trustworthy, and that it met their need for information (51–64%). At the same time, around one-third answered “to some extent” to these questions and about 10% replied they only found this information to be sufficient, understandable, and trustworthy either to a small extent or not at all. This finding is not unique to young people; many adults also have problems understanding and evaluating health-related information, as indicated in a large survey on health literacy [2]. About half of the adolescents responded that they were satisfied or very satisfied with the information provided (49%), while 39% answered that they were neither satisfied nor dissatisfied, and 12% were dissatisfied or very dissatisfied. Good information dissemination is important; both for the group that lies in the middle as well as for those who are clearly dissatisfied (about 10%). The information provided should therefore be adapted and thematically targeted to adolescents. If the information is understandable and trusted, it is more likely that they will act appropriately.

Most adolescents (42%, respectively) answered “to some extent” to the two questions “The provided information makes me feel safer” and “The provided information calms me down.”, while about a third stated that they were not reassured by the information they received. Thus, there is a significant proportion of adolescents who did not feel that the information they acquired made them more secure or reassured. There may be several reasons for this. In addition to the fact that there is a real reason to worry about the future due to the pandemic, it is also possible that the information was too extensive, overwhelming, unreliable or not quality-assured, which may have aggravated adolescents’ perception of the situation around Covid-19 [5, 7]. Secondly, there will always be a proportion of young people who are initially more vulnerable to anxiety and rumination, as indicated in the annual Norwegian study Ungdata (2019), in which 23% of high school students stated that they worry too much about things [29]. One recommendation might be to have an increased preparedness among low-threshold health services, which adolescents who are concerned about the situation can contact. It is also important to inform adolescents about reliable sources of information with quality-assured content to avoid misleading information that results in unnecessary worry. The provided information must not only inform, but also instruct and motivate adolescents to comply with the instructions for staying safe, such as recommendations of social distancing and not to meet in large groups. It is also important that the provided information be up-to-date, and that incorrect information be disproved [30].

### Impact on everyday life and concerns about infection

A total of 72% of adolescents stated that the pandemic affected their everyday life, and 21% reported that they were concerned about becoming infected with Covid-19. This concern may reflect more than concern for one’s own health, but also the fear of infecting others in high-risk groups. Girls worried more about this than boys, which corresponds to traditional gender differences, where girls tend to report higher levels of worry and anxiety [31–34], and to previous differences related to the Covid-19 pandemic [35]. A study conducted among adults in eight countries examined attitudes and behavior differences between genders related to the Covid-19 pandemic and found that women perceived Covid-19 to be a more serious health problem than men [36], which is in line with findings from our study of young people.

Concerns and emotions can affect how adolescents perceive information and how they assess risks. This in turn may affect their behavior [37]. Even when adolescents are well informed, they could rely more on their feelings about the situation, rather than the knowledge they have acquired. Thus, risk-based decisions, such as gathering at a party, can be difficult to influence, even when the provided information is adequate, understandable, and trustworthy [38]. On the other hand, just over half of adolescents reported that they were not concerned about becoming infected with Covid-19.

### Lack of information

When it came to information that adolescents felt was lacking, about one-third reported that they wanted more information on the school situation and exams, but also about how long the situation could last. Similarly, approximately one-third expressed concerns about infection and illness, such as quarantine rules and who belonged to high-risk groups. The last category was information about the future and concerns about lack of contact with friends and girl- and boyfriends. Many of the questions were related to the future, which reflects the needs of the adolescents who are exploring different options regarding work and education.

## Limitations

The Covid-19 pandemic is a situation that no one has experienced before, and no similar data have been obtained from adolescents in such an exceptional situation. There are several limitations to this study. Firstly, the sample is not representative of all Norwegian adolescents, as it is based on students from upper secondary school, and girls and pupils from the north are over-represented. Furthermore, the study link was spread through various online sources, which might affect the representativeness of the current findings. However, the Norwegian Media Authority’s annual report (2020) shows that 99% of 15–18-year-olds have their own mobile phone in addition to their own computer (85% to 99%) or tablet (45% to 51%) [25]. This could indicate that there might not be huge differences in internet access between adolescents in Norway. Since the study link was spread through online sources, it is not possible to calculate the response rate to the survey. Still, 377 adolescents did reply, and some provided more topics about which they felt they needed more information; many of the questions they asked may also be held by other adolescents. Since the study link was spread through online sources, it is also possible that an adolescent completed the survey more than once. However, no incentive was offered to complete the survey, making this less likely. The response options regarding sources of information in the survey were not defined and could be subject to various interpretations by the participants. For example, the category “school” does not imply that that the information was obtained through a teacher-student lecture; it could also be obtained through internet-based resources from school, from a peer, or from a flyer from school. At the time we conducted the survey, schools were closed, and it may have been less clear what that response option meant. That is, the students could have received information either directly from their teacher in video sessions or in written form. Furthermore, the question asked where adolescents *got* their information on Covid-19, which does not imply that adolescents were actively seeking out information on specific channels. Another limitation is due to the nature of the survey, as most of the questions were closed. The current study could be supplemented by future qualitative studies that would enhance our understanding of some of the findings. Also, this study did not test adolescents' knowledge of Covid-19 based on where they were getting their information, which would be interesting to explore in future research. The current study did not examine what adolescents did with the information they reported to receive. Future research could also examine how this information might have influenced their behavior.

## Conclusions

Many health professionals, as well as the Norwegian Institute of Public Health, have argued that the pandemic may have severe consequences for children and young people [39, 40]. Efficient dissemination of information is an important part of public health work; it has been found to play a huge role in reducing infections, and places a low to no burden on children and adolescents [1]. The results of this survey provide important knowledge to those working with adolescents in schools and in various child and adolescent services. The results could also provide important information to authorities as to what information channels can be used to reach adolescents, and about which topics they seek information. In summary, the results of this study indicate that there is a need to increase the proportion of adolescents who are satisfied with the information provided about the Covid-19 pandemic. This study shows that adolescents want more information about the school situation, as well as virus-related and future-related topics. Measures that contribute to providing personalized information are recommended, as they give adolescents an opportunity to ask questions and get answers to their specific questions. In order to respond to these specific questions, and to reach as many adolescents as possible, another recommendation might be to hold a press conference or similar event for adolescents, as was done for children, on a national level. Some information is more specific to each school or county and should be disseminated locally. In this study, most adolescents rated newspapers/online newspapers as the best source of information, and thus it may be useful to publish more information tailored to adolescents in these media. The internet is the source used by most youth to get information about the situation. Digital platforms, where adolescents can ask questions and get quality-assured answers, can be important sources for reaching adolescents in an exceptional situation such as the Covid-19 pandemic.

## Data Availability

Replication data for “Adolescents Experiences of the Information they received about the Coronavirus (Covid-19) in Norway” (view at https://dataverse.no/dataset.xhtml?persistentId=doi:10.18710/OSAYBD) was published in UiT Open Research Data (view at https://dataverse.no/dataverse/uit).
